# Polymer Composite-Based Triboelectric Nanogenerators: Recent Progress, Design Principles, and Future Perspectives

**DOI:** 10.3390/polym17141962

**Published:** 2025-07-17

**Authors:** Geon-Ju Choi, Sang-Hyun Sohn, Se-Jin Kim, Il-Kyu Park

**Affiliations:** Department of Materials Science and Engineering, Seoul National University of Science and Technology, Seoul 01811, Republic of Korea; geonju0927@naver.com (G.-J.C.);

**Keywords:** polymer composite, triboelectric nanogenerators (TENGs), inorganic fillers, nanofibers

## Abstract

The escalating consumption of fossil fuels and the rapid development of portable electronics have increased interest in alternative energy solutions that can sustainably self-power wearable devices. Triboelectric nanogenerators (TENGs), which convert mechanical energy into electricity through contact electrification and electrostatic induction, have emerged as a promising technology due to their high voltage output, lightweight design, and simple fabrication. However, the performance of TENGs is often limited by a low surface charge density, inadequate dielectric properties, and poor charge retention of triboelectric materials. To address these challenges, recent research has focused on the use of polymer composites that incorporate various functional fillers. The filler materials play roles in improving dielectric performance and enhancing mechanical durability, thereby boosting triboelectric output even in harsh environments, while also diminishing charge loss. This review comprehensively examines the role of polymer composite design in TENG performance, with particular emphasis on materials categorized by their triboelectric polarity. Tribo-negative polymers, such as PDMS and PVDF, benefit from filler incorporation and phase engineering to enhance surface charge density and charge retention. By contrast, tribo-positive materials like nylon and cellulose have demonstrated notable improvements in mechanical robustness and environmental stability through composite strategies. The interplay between material selection, surface engineering, and filler design is highlighted as a critical path toward developing high-performance, self-powered TENG systems. Finally, this review discusses the current challenges and future opportunities for advancing TENG technology toward practical and scalable applications.

## 1. Introduction

With the increasing global reliance on fossil fuels and the accelerating rate of their consumption, the need for alternative and sustainable energy sources has become more urgent than ever. In parallel, the rapid development of portable and wearable electronic devices has intensified the demand for continuous and reliable power supply systems. Although advances in battery technology have extended the operating time of electronic devices by increasing energy density, their inherent limitations, such as finite capacity, considerable weight and volume, and the necessity for periodic recharging, remain major obstacles, especially in mobile and remote applications, for the realization of the Internet of Things.

Energy harvesting technologies offer a compelling solution by converting ambient energy sources, such as mechanical motion, heat, light, and electromagnetic waves, into usable electrical energy [1]. These technologies are being extensively explored as potential alternatives or supplements to batteries, particularly for enabling self-powered wearable systems and autonomous electronics that require uninterrupted power without human intervention [2]. By capturing otherwise wasted energy and utilizing it efficiently, energy harvesters contribute not only to powering devices sustainably but also to advancing the broader transition toward cleaner and more energy-efficient technologies.

Among various technologies that convert mechanical energy into electricity, the triboelectric nanogenerator (TENG) has gained significant attention for its combination of high voltage output, lightweight design, and structural simplicity [3,4,5,6,7]. Based on the coupling of the triboelectric effect and electrostatic induction, TENGs generate electricity through repeated contact and separation between two materials with different electron affinities. This principle generates power from mechanical movements such as human motion [8,9,10,11], flow of substances [12,13], or other mechanical activities [14,15,16]. Their compatibility with polymer-based materials allows for highly flexible, low-cost, and easily fabricated energy harvesters, making them ideal for next-generation wearable electronics and self-powered sensing systems.

**Figure 1:**
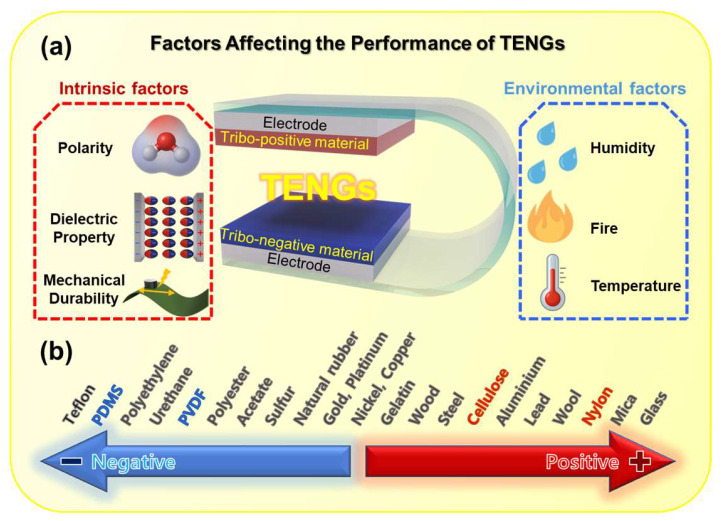
(**a**) Schematic illustration of factors influencing triboelectric nanogenerators (TENGs). (**b**) Triboelectric series.

However, despite these advantages, the output performance of TENGs is still limited by various factors, such as a low surface charge density, weak charge retention, and the suboptimal dielectric properties of base materials [17,18]. To make matters worse, the durability and reliability of TENG devices raise issues that need to be solved for practical application under harsh conditions, such as severe friction, elevated temperature, and humid environments. During the operation of TENG devices, mechanical friction between the contact surfaces of the charge-generating materials inevitably occurs, which damages the materials. To address these issues, recent studies have focused on designing polymer composite materials that incorporate functional fillers, such as dielectric nanoparticles, conductive nanomaterials, or surface modifiers, to enhance the electrical and mechanical properties of triboelectric layers (Figure 1a).

This review aims to present a comprehensive overview of polymer composite-based TENGs, with a particular emphasis on material strategies categorized by triboelectric polarity. We highlight recent advancements in tribo-positive and tribo-negative composite design, analyze their structure–property–performance relationships, and discuss current challenges and future research directions toward high-performance, self-powered systems. Building upon these insights, this review aims to guide future research by proposing strategic design principles for optimizing polymer composites, promoting multifunctionality, and enhancing environmental robustness, thereby contributing to the practical deployment of flexible and durable TENG devices.

## 2. Factors Affecting the Performance of TENGs

The performance of a TENG is influenced by multiple interrelated factors that collectively determine its efficiency in converting mechanical energy into electrical energy (Figure 1a). Among these, the choice of triboelectric materials is a primary consideration, as it directly affects the amount of surface charge generated during contact and separation cycles [17,18]. Materials are typically selected based on their position in the triboelectric series, which categorizes substances according to their tendency to donate or accept electrons, as shown in Figure 1b. A larger difference in electron affinity between the paired materials induces a stronger triboelectric effect, which consequently results in a higher surface charge density. However, it is important to note that the triboelectric series is empirical, and its ordering can vary depending on the experimental conditions, measurement methods, and material-specific parameters. Factors such as surface chemistry, roughness, contamination, moisture, and ambient humidity can significantly influence the actual triboelectric behavior of materials in practical applications. The triboelectric charge density (*σ*), defined as the amount of transferred charge (*Q*) per unit contact area (*S*), is expressed as follows:

(1) σ=Q/S

Commonly used materials include electronegative polymers such as PTFE and PVDF and electropositive materials such as nylon or aluminum, respectively. The combination of materials from opposite ends of the triboelectric series maximizes charge separation, leading to enhanced electrical output.

Another crucial factor is the effective contact surface area between triboelectric layers [19,20,21,22,23,24,25,26]. By enlarging the contact surface, the amount of charge that can be transferred increases during each operation cycle because the total charge generated (*Q_Total_*) is proportional to the surface charge density (*σ*) and the specific surface area (*S*). To maximize the specific surface area without increasing device size, micro- and nano-structuring techniques are frequently employed. Surface engineering approaches, such as the introduction of micropatterns, nanopillars, or nanowires, significantly increase the effective contact area, thereby boosting charge generation efficiency. Additionally, the fabrication of nanofibrous membranes via a novel process such as electrospinning provides a high surface-to-volume ratio, further enhancing the effective surface area for charge accumulation.

Beyond material selection and surface structuring, environmental and mechanical factors also play pivotal roles in determining TENG performance. For instance, in high-humidity environments, the increased presence of water molecules in the air accelerates the dissipation of triboelectric charges, thereby reducing the surface charge density and degrading the overall performance of TENGs [27,28,29,30]. To mitigate such environmental degradation, various approaches have been explored, including the use of humidity-resistant polymer matrices, the incorporation of hydrophobic nanofillers, and additives that have high thermal conductivity. These strategies are discussed in detail in subsequent sections. Likewise, ambient temperature can influence electron mobility and surface conductivity. Moderate heat may enhance performance, while excessive heat may lead to charge leakage or dielectric breakdown [31,32].

The mechanical input conditions, including the applied contact force and the frequency of operation, are equally critical. An increased contact force not only enhances the physical intimacy at the interface between triboelectric layers but also intensifies the frictional interaction, thereby amplifying the triboelectric effect and increasing the amount of charge generated during each cycle [33]. In addition, increasing the frequency of contact and separation cycles raises the rate of charge accumulation [14,34]. Since surface charges on triboelectric materials tend to dissipate over time due to environmental influences, such as the presence of water molecules in humid air, more frequent mechanical cycling can help to compensate for this natural charge loss. As a result, increasing the operational frequency allows the device to achieve a higher saturated surface charge density, thereby enhancing its overall energy output. Taken together, the optimization of material pairing, surface architecture, environmental stability, and mechanical dynamics offers a comprehensive strategy for maximizing TENG output and adapting the technology to a wide range of applications, from wearable electronics to autonomous wireless sensing systems.

## 3. Tribo-Negative Materials

Tribo-negative materials play a central role in the construction of triboelectric nanogenerators, as they have a strong tendency to attract and retain electrons through the process of contact electrification. These materials are located at the negative end of the triboelectric series and are characterized by their high electron affinity, which enables them to accumulate negative surface charges when brought into contact with more electropositive materials. Common tribo-negative polymers include fluorinated materials such as polytetrafluoroethylene (PTFE), polyvinylidene fluoride (PVDF), and fluorinated ethylene propylene (FEP). These materials have been widely used due to their high electronegativity, strong electrical insulation properties, and chemical stability, which make them suitable for effective charge generation. In addition to fluorinated polymers, polydimethylsiloxane (PDMS) has also been extensively used as a tribo-negative material, particularly in flexible and wearable energy harvesting devices. Although its electronegativity is moderate compared to fluorinated polymers, PDMS offers exceptional advantages, such as high mechanical flexibility, ease of processing, and compatibility with micro- and nano-structuring techniques. These features make it a highly versatile platform for constructing composite triboelectric layers with tailored mechanical and electrical properties. Recent research has focused on improving the performance of these tribo-negative materials by incorporating them into composite structures. This includes the introduction of high-permittivity dielectric fillers, conductive nanomaterials, and surface modification techniques that enhance charge generation, storage stability, and durability.

### 3.1. Polydimethylsiloxane (PDMS): Flexible and Structurally Tunable Polymer

PDMS has emerged as one of the most popular tribo-negative polymers for TENGs owing to its strong tendency to gain electrons (high electronegativity). It offers several key advantages that make it an ideal triboelectric layer. First, PDMS is highly flexible and stretchable, enabling devices to withstand bending, stretching, and deformation without cracking [35]. The unique molecular structure of PDMS is the primary reason for these properties. The backbone of PDMS consists of repeating Si–O–Si units, which possess long bond lengths, wide bond angles, and low rotational energy barriers, resulting in exceptional chain mobility. In addition, PDMS exhibits a very low glass transition temperature (T*_g_* ≈ −125 °C), allowing it to remain in a rubbery, elastic state at room temperature. The presence of small, nonpolar methyl side groups (–CH_3_) minimizes intermolecular interactions and suppresses crystallization, further enhancing its softness and deformability. This combination of structural characteristics leads to an amorphous, highly flexible material. This elasticity is crucial for wearable and biomechanical TENG applications. For example, thin PDMS films have been applied to moving parts, like hands and knees, to detect motion while generating electricity. Secondly, PDMS demonstrates outstanding processability because it behaves as a liquid elastomer that can be easily molded and micro-patterned [35,36]. Soft lithography or template-based molding processes using textured substrates like sandpaper enable the fabrication of micro- or nano-textured surfaces. These structures significantly enhance TENG performance by enlarging the effective contact area. Third, PDMS is an electrical insulator with a moderate dielectric constant of approximately 2.7~3.0 [37]. Its insulating nature enables it to retain triboelectric charges on the surface without rapid leakage, maintaining the charge separation required for TENG operation. Although the relative permittivity of pristine PDMS is relatively low, it can be tuned and increased in various ways. By adjusting the curing ratio, its stiffness and polar group concentration can be modified consistently. In addition, incorporating high-dielectric particles as filler into PDMS materials is a widely employed strategy to improve charge generation. PDMS exhibits excellent compatibility with composite formation, as its matrix can readily accommodate various nanoparticles, nanofibers, or functional additives. Blending high-dielectric permittivity ceramics or conductive nanostructures into PDMS increases the effective dielectric constant of the composite, which in turn increases the surface charge density and electrical output. Numerous studies have reported that embedding micro- and nano-scale fillers in PDMS films leads to significant performance improvements. Lastly, PDMS is known for its low cost, long-term chemical stability, biocompatibility, and even optical transparency. These properties make PDMS-based TENGs highly versatile, enabling their use in bio-integrated, wearable, and transparent electronics while maintaining high-performance energy harvesting capabilities. Despite these favorable properties, pristine PDMS has its limitations. Under prolonged cyclic stress, UV exposure, or high temperatures, PDMS can undergo mechanical fatigue, leading to surface microcracks, loss of elasticity, and reduced TENG performance. To address these durability issues, recent studies have incorporated functional fillers into PDMS matrices. These additives, previously introduced to enhance dielectric properties, also significantly contribute to improving the long-term durability of PDMS under mechanical and thermal stress.

**Figure 2:**
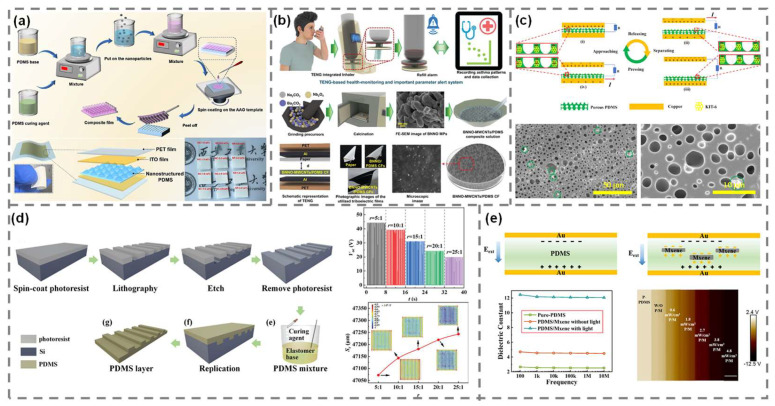
Design strategies for PDMS-based TENGs. (**a**) Nanopillar- and nanocone-structured SrTiO_3_/PDMS films. Reproduced with permission [38]. Copyright © 2024, American Chemical Society. (**b**) BNNO-MWCNTs/PDMS composite. Reproduced with permission [39]. Copyright © 2024, Wiley-VCH GmbH. (**c**) Silica/PDMS composite. Reproduced with permission [40]. Copyright © 2025, Elsevier. (**d**) Effect of PDMS base/agent ratio. Reproduced with permission [42]. Copyright © 2022, Wiley-VCH GmbH. (**e**) PDMS/MXene composite. Reproduced with permission [43]. Copyright © 2021, Elsevier.

Recent studies have demonstrated that incorporating functional fillers into PDMS matrices can significantly enhance the triboelectric performance of TENGs by enabling dielectric enhancement, charge trapping, and improvements in contact efficiency. In one example, Meng et al. used an anodized aluminum oxide (AAO) template to fabricate PDMS nanocone films, embedding 9 wt% SrTiO_3_ nanoparticles. This structural and compositional hybridization resulted in an open-circuit voltage of 130 V and a short-circuit current of 1.4 μA, outperforming flat PDMS by maximizing the contact area and local electric field concentration (Figure 2a) [38]. Similarly, a PDMS composite incorporating 0.25 wt% Ba_2_NaNb_5_O_15_ (BNND) achieved an output voltage near 300 V and a current of 8.5 μA, driven by the permittivity and residual polarization of filler (Figure 2b) [39]. The addition of a small amount of multi-walled carbon nanotubes (MWCNTs) into the BNNO-PDMS matrix further improved charge transport and stability.

Charge trapping is another effective mechanism for improving TENG output. Mao et al. introduced 2 wt% mesoporous KIT-6 silica into PDMS, creating many internal trap sites for electrons (Figure 2c) [40]. This composite generated a power density of 4.37 W/m^2^, which was approximately 6.5-times higher than that of pure PDMS and maintained improved performance under both low and high mechanical loads [40]. Zhu et al. implemented an ultra-low concentration (0.005 wt%) of Ag nanowires into PDMS, forming nanoscale capacitive gaps between wires [41]. When combined with a rough surface prepared using sandpaper as a template, the resulting device achieved an instantaneous power density of approximately 162 mW/m^2^, which represented a nearly 700-fold enhancement over flat pristine PDMS (0.23 mW/m^2^).

Sun et al. focused on optimizing the polymer matrix itself, tuning the PDMS curing ratio to control the polar group density and elastic modulus. They found that decreasing the base-to-curing agent ratio increased the dielectric constant and improved energy output despite a slight reduction in contact area (Figure 2d) [42]. Finally, a composite of PDMS and MXene nanosheets was used to construct a stretchable TENG that exhibited significantly enhanced performance (Figure 2e) [43]. The addition of MXene increased the dielectric constant from 2.65 to 5.74 and shifted the surface potential to a more negative value, as confirmed by Kelvin probe force microscopy (KPFM) analysis, indicating improved charge retention capability. The device achieved an open-circuit voltage of 453 V and a short-circuit current of 131 μA, surpassing the performance of pristine PDMS. This improvement was attributed to the high electronegativity and abundant terminal functional groups of MXene, which facilitated stronger triboelectric interactions and more efficient charge separation.

In summary, PDMS serves as a versatile and tunable platform for TENG applications, particularly when used as a composite with functional fillers. Many previous studies have demonstrated that combining structural modifications, dielectric enhancement, and charge retention strategies can effectively overcome the intrinsic limitations of pristine PDMS. Table 1 summarizes representative studies utilizing PDMS-based composites for TENG applications, highlighting each process and their corresponding performance outcomes. These advances indicate that rational composite design not only improves triboelectric output but also expands the application potential of PDMS-based nanogenerators in wearable and flexible energy harvesting systems. However, PDMS has several process limitations that need to be addressed, as it is very challenging to manufacture high-surface-area nanofibers through electrospinning. As a result, performance enhancement mainly relies on surface nanopatterning technology, which is difficult to implement uniformly over large areas and may have limitations in maintaining long-term structures. These issues can hinder large-scale production and commercialization, especially in applications that require high resolution and high reproducibility. On the other hand, PDMS possesses properties such as mechanical flexibility, biocompatibility, and optical transparency, making it suitable for applications like skin-attachable sensors, wearable electronics, and stretchable energy harvesters. Since these applications prioritize wearability, deformability, and biocompatibility over maximum performance, they offer a practical direction that leverages the unique advantages of PDMS.

### 3.2. Polyvinylidene Fluoride (PVDF): Phase Engineering

Among tribo-negative polymers, PVDF has received considerable attention due to its distinctive semi-crystalline structure and the ability to undergo phase transformations that significantly affect its electrical properties. PVDF can exist in several crystalline phases, including the non-polar *α*-phase, the polar *β*-phase, and less common *γ*- and *δ*-phases. Among them, the *β*-phase is of particular interest for TENG applications because it features highly aligned molecular dipoles that enhance both piezoelectric and triboelectric responses [44].

The molecular structure of the *β*-phase is characterized by an all-trans planar zigzag conformation, which aligns the carbon-fluorine dipoles along the same direction, thereby increasing the net dipole moment. This structural arrangement results in a higher surface charge density and more efficient energy conversion during contact electrification. Therefore, a main strategy in PVDF-based TENG research involves promoting the formation of the *β*-phase within the polymer matrix.

**Figure 3:**
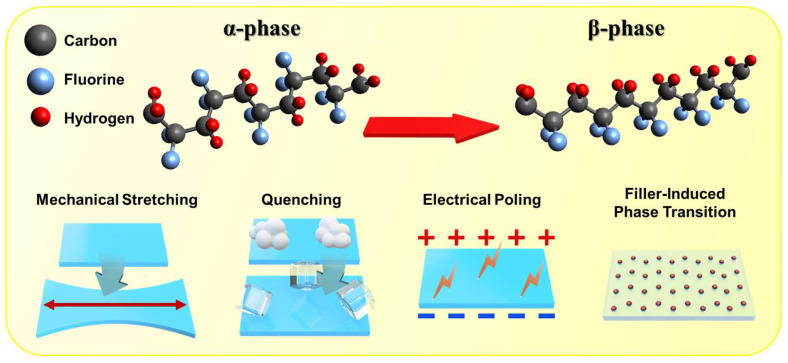
Schematic illustration of the *α*-to-*β*-phase transition in PVDF through mechanical stretching, quenching, electrical poling, and filler incorporation to enhance dipole alignment and TENG performance.

Various methods have been employed to increase the *β*-phase content in PVDF. These include mechanical stretching [45,46], which physically aligns the polymer chains; electrical poling [47], which orients the dipoles through an external electric field; and rapid quenching from high temperature [48], which kinetically traps the chains in the desired conformation (Figure 3). Additionally, chemical approaches such as copolymerization with trifluoroethylene (PVDF-TrFE) or poly(methyl methacrylate) and the incorporation of nanofillers or additives can facilitate the nucleation and stabilization of the *β*-phase [49]. These additives, including BaTiO_3_ nanoparticles, carbon-based nanomaterials, and ionic liquids, have been shown to interact with PVDF chains and promote dipole alignment.

Beyond phase control, composite strategies also contribute to improved triboelectric performance. By incorporating dielectric or conductive fillers into PVDF, researchers have achieved synergistic enhancements in surface charge generation, mechanical durability, and environmental stability. The interplay between phase engineering and composite design makes PVDF a uniquely versatile tribo-negative material with broad potential in both wearable and structural energy harvesting applications.

#### 3.2.1. PVDF-Carbon Composites

Carbon-based nanomaterials, such as graphene, carbon nanotubes (CNTs), and fullerenes (C_60_), have emerged as promising functional nanofillers for enhancing the performance of polymer-based TENGs. These materials possess unique combinations of high electrical conductivity, large specific surface area, mechanical robustness, and chemical stability, making them well suited for integration into flexible polymer matrices [50]. When incorporated into PVDF, carbon nanofillers contribute to performance improvement through several mechanisms. First, they provide efficient charge transport pathways within the composite, reducing internal charge recombination and facilitating the movement of induced charges to external electrodes. Secondly, interfacial interactions between carbon nanofillers and the PVDF matrix can promote the nucleation of the polar *β*-phase, thereby enhancing dipole alignment and surface charge density. Additionally, their high aspect ratio and nanoscale roughness enhance the interfacial area between triboelectric layers, further boosting contact electrification efficiency.

The multifunctional nature of carbon-based fillers allows the simultaneous enhancement of electrical, mechanical, and surface properties, making them particularly attractive for designing high-performance PVDF-based composites for TENG applications. Among the various carbon-based nanofillers investigated for enhancing PVDF-based TENGs, graphene and its derivatives have received particular attention due to their structural and electronic characteristics that support multiple performance enhancement mechanisms. Rather than serving merely to improve structural stability, graphene-based materials play active roles in modulating the crystalline phase, improving interfacial charge behavior, and stabilizing the performance of TENGs over time. 

**Figure 4:**
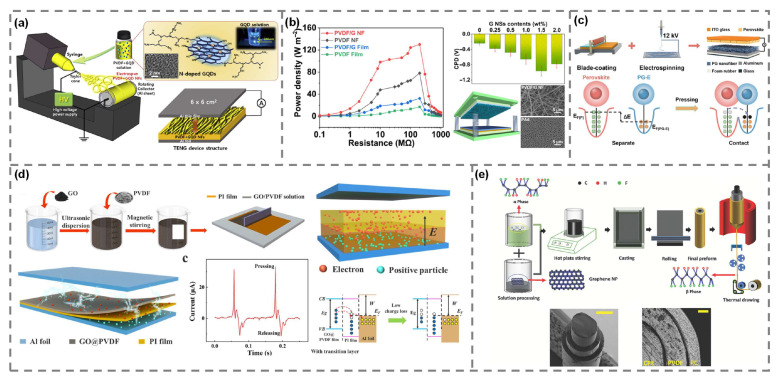
Design strategies for PVDF/carbon-based composite TENGs. (**a**) PVDF/graphene quantum dot composite nanofiber. Reproduced with permission [51]. Copyright © 2019, Elsevier. (**b**) PVDF/graphene nanosheet composite nanofiber. Reproduced with permission [52]. Copyright © 2021, Elsevier. (**c**) PVDF/graphene nanofiber. Reproduced with permission [53]. Copyright © 2024, Elsevier. (**d**) PVDF/graphene oxide composite. Reproduced with permission [54]. Copyright © 2025, Elsevier. (**e**) PVDF/graphene nanoplatelet. Reproduced with permission [55]. Copyright © 2024, Wiley-VCH GmbH.

One of the most notable effects of graphene incorporation is the promotion of the polar *β*-phase in PVDF. The π-conjugated surface of graphene offers nucleation sites that promote the alignment of PVDF chains into the all-trans configuration. This improves dipole alignment and increases surface charge density. *β*-phase enhancement has been consistently reported in most PVDF-graphene composite structures, regardless of the fabrication method, like electrospinning or screen printing [51,52,53,54,55]. By contrast, PVDF-hexafluoropropyl copolymer (PVDF-HFP)-based composite [56], which already possesses a high intrinsic β-phase content, showed a slight decrease in the *β*-phase fraction with increasing graphene content. A similar trend was observed in graphene quantum dot (GQD)-PVDF-based composite (Figure 4a) [51]. The results showed that excessive loading of GQD nanofiller may induce aggregation and hinder dipole alignment, ultimately reducing crystallinity rather than improving it. In addition to crystalline phase modulation, several studies have reported that the incorporation of graphene also leads to an improvement in the dielectric properties of PVDF-based composites, which can further enhance TENG performance. A higher dielectric constant increases the ability of the material to store electrostatic charges at the interface, thereby facilitating more efficient charge induction and transfer during contact electrification.

In PVDF composite incorporated with graphene oxide (Figure 4d) [54], the dielectric constant increased by approximately 51%, resulting in significant improvements in charge density and output power. However, at higher graphene oxide contents, the rising electrical conductivity promoted internal charge recombination, ultimately reducing the output performance. A similar trend was observed in PVDF-HFP/graphene composite, where the dielectric constant initially increased due to interfacial polarization, but excessive graphene loading caused dielectric loss and filler agglomeration, leading to a degradation in output power [56]. These results highlight the dual role of graphene in enhancing interfacial polarization and tuning electric properties. To verify the electronic effects underlying the performance enhancements attributed to crystalline and dielectric modifications, several studies employed KPFM measurement to quantitatively assess surface potential changes in PVDF/graphene composites. This analytical approach enabled researchers to observe that the incorporation of graphene leads to a more negative surface potential. This shift reflects an increase in electron affinity and an enhanced ability of the material to retain negative charges, both of which contribute to tribo-negative behavior. For example, in a PVDF composite containing graphene nanosheets (Figure 4b), the contact potential difference decreased from −0.24 V in pure PVDF to −0.96 V for 1.5 wt% graphene embedding composite [52]. This change resulted in a substantial increment in TENG performance. A similar result was observed in PVDF composite with graphene oxide and a polyimide interlayer, where the surface potential reached −5.78 V after tribo-charging [54]. Additionally, in a separate study, PVDF/graphene composite was tested against a perovskite counter layer (Figure 4c) [53]. This structure showed a lower surface potential and reduced work function, which were consistent with improved charge transfer performance. These results confirm that triboelectric performance enhancement arises not only from structural and dielectric modifications but also from an improved electron-accepting nature of the composite surface, as demonstrated through KPFM analysis. A summary of the fabrication process, performance enhancement factors, and output performance reported in these studies is provided in Table 2.

#### 3.2.2. Non-Carbon Additive-Based PVDF Composite

In addition to carbon-based nanofillers, numerous studies have explored the incorporation of non-carbon materials into PVDF to enhance TENG performance. Notable examples include ferroelectric BaTiO_3_ [57], magnetic Fe_3_O_4_ [58], layered-double hydroxides (LDH) [59], two-dimensional nanomaterials (such as MXene and MoS_2_) [60,61], metal–organic frameworks (MOFs) [62], and metallic nanostructures (Ag nanowires) [63]. These diverse additives consistently promote the formation of the electroactive *β*-phase and significantly improve the dielectric properties and charging performances of the composites. Consequently, PVDF nanocomposites with such nanofillers exhibit greater polarization and surface charge density, leading to improved TENG performance. Notably, the underlying enhancement mechanisms are largely analogous to those observed with carbon-based nanofillers such as graphene and CNTs.

**Figure 5:**
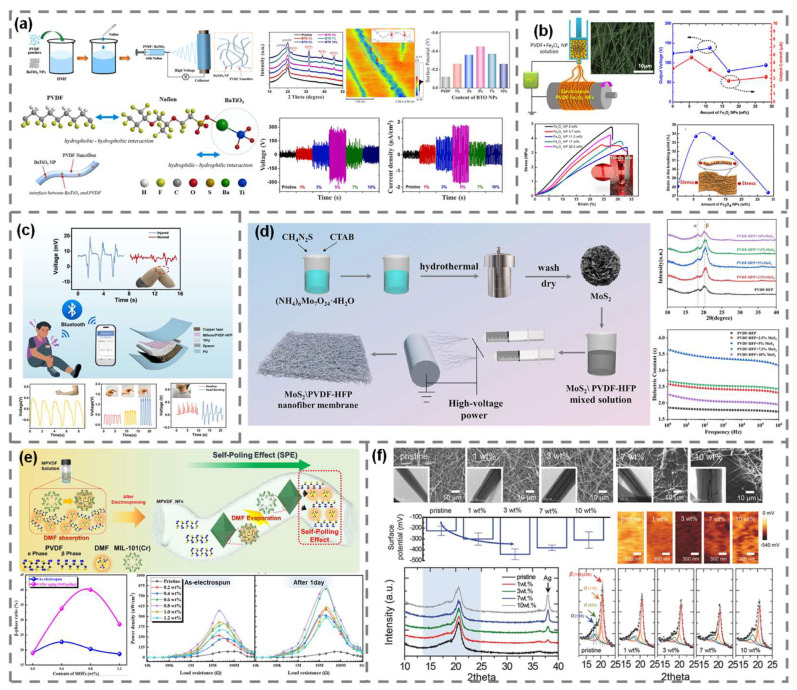
Design strategies for PVDF/non-carbon-additive-based composite TENGs. (**a**) PVDF/BaTiO_3_ composite nanofiber. Reproduced with permission [57]. Copyright © 2023, Elsevier. (**b**) PVDF/Fe_3_O_4_ composite nanofiber. Reproduced with permission [58]. Copyright © 2018, American Chemical Society. (**c**) PVDF-HFP/MXene composite fiber. Reproduced with permission [60]. Copyright © 2024, Elsevier. (**d**) PVDF/MoS_2_ composite. Reproduced with permission [61]. Copyright © 2025, Elsevier. (**e**) PVDF/MOF composite nanofiber. Reproduced with permission [62]. Copyright © 2023, Elsevier. (**f**) PVDF/AgNW composite nanofibers. Reproduced with permission [63]. Copyright © 2017, WILEY-VCH Verlag GmbH & Co. KGaA, Weinheim, Germany.

Metal oxide nanoparticles are among the most widely investigated non-carbon fillers for PVDF-based TENGs due to their inherent dielectric and interfacial properties. Many metal oxides possess high relative permittivity, ferroelectricity, or charge trapping capabilities, allowing them to influence the crystalline phase behavior of PVDF and enhance its dielectric response. Their tunable surface chemistry and nanoscale dispersibility make them ideal for incorporation into electrospun or solution-processed polymer composites. Among them, BaTiO_3_ and Fe_3_O_4_ nanoparticles represent two functionally distinct but effective oxide additives. BaTiO_3_, a classic ferroelectric material with a high dielectric constant, was blended with PVDF and electrospun into nanofibrous mats (Figure 5a) [57]. Nafion was used as a dispersion agent to enhance compatibility. The embedded BaTiO_3_ nanoparticles facilitated stress-mediated nucleation of the polar *β*-phase, increasing the dielectric constant of the composite nanofibers, which in turn enhanced the surface charge density. As a result, the TENG achieved a peak open-circuit voltage of approximately 307 V and a current density of 1.8 μA/cm^2^ at 5 wt% loading, delivering a maximum power density of 1.12 mW/cm^2^, which was more than six times that of pristine PVDF nanofibers.

Fe_3_O_4_, a magnetic metal oxide with moderate dielectric properties, was similarly electrospun with PVDF to form nanofibers. The optimized composite nanofibers, containing 11.3 wt% Fe_3_O_4_ nanoparticles, exhibited enhanced *β*-phase crystallinity and behaved similarly to electrets, improving long-term charge storage (Figure 5b) [58]. This enabled a TENG performance of 138 V and 5.68 μA, representing 11% and 77% increases in voltage and current, respectively, compared to pristine PVDF nanofibers. However, excessive loading of Fe_3_O_4_ nanoparticles resulted in agglomeration, which degraded interfacial contact and output performance. In addition to energy harvesting, the incorporation of magnetic Fe_3_O_4_ nanoparticles conferred multifunctional properties to the composite, including enhanced electromagnetic interference shielding efficiency due to magnetic loss and interfacial polarization, as well as improved mechanical strength, which benefits structural integrity and device durability in practical applications.

LDHs are two-dimensional anionic clays composed of positively charged metal hydroxide layers and charge-compensating interlayer anions. Their high aspect ratio, surface hydroxyl groups, and interfacial polarization capability make them attractive candidates for polymer nanocomposites, particularly in tuning crystallinity and dielectric behavior. When ZnAl-LDH was incorporated into PVDF via solution blending and casting, a spontaneous transition from α-phase to *β*-phase occurred, even without additional electrical poling or stretching processes [59]. This effect was attributed to strong hydrogen bonding and interfacial interactions between LDH platelets and PVDF chains, which acted as nucleation sites for electroactive crystal growth. Additionally, the inherent dielectric properties of LDHs significantly enhanced the permittivity of the composites. The optimized composite structures, containing 20 wt% LDH, showed an open-circuit voltage of approximately 230.6 V and a current density of 5.6 mA/cm^2^, corresponding to a power density of 0.43 mW/cm^2^. This represented approximately a 6.8-fold increase in voltage and a 5.9-fold increase in current compared to pristine PVDF. Notably, the composite maintained high optical transparency, enabling its use in transparent TENGs, which is a property rarely achieved with other high-performance fillers.

Two-dimensional (2D) nanomaterials with high surface electronegativity, such as MXene and MoS_2_, have emerged as highly effective functional fillers for PVDF-based TENGs [60,61]. Their ultrathin sheet structures with abundant surface terminations facilitate strong interfacial interactions and dipole alignment, which are essential for enhancing the electroactive *β*-phase content, surface charge density, and dielectric constant of PVDF composites. MXene, specifically Ti_3_C_2_T_x_, exhibits excellent surface functionality owing to its abundant electronegative groups (-F, -OH, etc.). When incorporated into PVDF-HFP via electrospinning, MXene significantly promoted *β*-phase crystallization due to its electronegative nature and interfacial compatibility. The optimized composite containing 2.5 wt% MXene delivered a peak-to-peak open-circuit voltage of approximately 160 V, attributed to improved dipole orientation, an increased dielectric constant, and reduced surface potential (Figure 5c) [60].

MoS_2_, another widely studied 2D material, was embedded into PVDF-HFP nanofibers to exploit its layered structure and moderate surface reactivity. At an optimal loading of 5 wt%, the composite showed substantial increases in both the *β*-phase fraction and dielectric permittivity (Figure 5d) [61]. The resulting TENG performance reached 208 V and 31 mA, corresponding to a power density of 1.42 W/m^2^. These values were approximately 1.7-times higher in voltage and nearly 2.8-times higher in current than those of pristine PVDF. These two studies demonstrated that electronegative 2D nanomaterials were capable of tuning both the structural and dielectric properties of PVDF-based composites. Their synergistic influence on dipole alignment, interfacial charge trapping, and dielectric response makes them promising multifunctional additives for TENG applications.

MOFs, with their unique properties of high surface area, tunable pore environments, and rich functional groups, are emerging as promising materials for TENGs. The ability of MOFs to adsorb guest molecules and modulate interfacial polarization has sparked a wave of interest in their application as active fillers in TENGs. Incorporating MIL-101(Cr) MOF nanoparticles into PVDF nanofibers via the electrospinning process has shown significant potential. The porous structure of the MOF enables enhanced charge trapping, facilitating the spontaneous formation of the polar *β*-phase. This is attributed to a combination of physical confinement effects, interfacial interactions, and a self-poling phenomenon (Figure 5e) [62]. The latter is driven by residual solvent molecules adsorbed in the pores of the MOF, which induce a precise local dipole alignment even after the electrospinning process. The residual solvent acts as a solvent-annealing agent. PVDF composite nanofibers containing 0.8 wt% MOF exhibited an open-circuit voltage of 414 V and a short-circuit current of 14.6 μA, resulting in a power output of 568.8 μW/cm^2^. These values represented more than a 3.7-times improvement in voltage and over a 4.1-times improvement in current compared to pristine PVDF. Furthermore, after a 24 h resting period, additional enhancement was observed due to post-process dipole rearrangement, supporting the presence of solvent-induced polarization effects [62].

Silver nanowires (AgNWs) are highly conductive one-dimensional metallic nanostructures with high aspect ratios and large surface areas. When integrated into polymer composites, they can form conductive networks and act as efficient charge-trapping sites, while also modifying the local surface potential [63]. These properties make AgNWs attractive additives for enhancing charge separation and transport properties in TENGs. AgNWs were embedded within PVDF nanofibers through electrospinning, forming a distributed conductive network within the polymer matrix (Figure 5f). At an optimal loading of 3 wt% AgNWs, the nanocomposite exhibited a peak open-circuit voltage of 240 V under a 5 N mechanical input force. This represented a 1.54-fold increase compared to pristine PVDF nanofibers, which showed a voltage of 156 V under the same conditions. The enhancement was attributed to a combination of various factors, including increased *β*-phase crystallinity, an elevated dielectric constant, and a reduction in surface potential due to the local electrostatic field generated by the AgNWs. However, further increasing the AgNW content beyond 3 wt% led to significant agglomeration of the nanofiller, which deteriorated the nanofiber morphology and reduced the effective contact area. This resulted in decreased TENG performance, highlighting the importance of controlling nanofiller dispersion.

The studies reviewed in this section collectively highlight the critical role of phase engineering in enhancing the performance of PVDF-based TENGs. Table 3 summarizes the process and performance outcomes reported in the reviewed studies. As a semicrystalline polymer, PVDF demonstrates strong potential for energy harvesting applications due to the high electronegativity of its polar *β*-phase. A variety of composite strategies, particularly the incorporation of functional fillers, have been shown to significantly promote the formation of the *β*-phase while also improving dielectric properties, surface potential, and charge retention. Carbon-based nanofillers, such as graphene, fullerene, and their derivatives, promote *β*-phase nucleation and enhance surface polarity through interfacial interactions and electron affinity. Similarly, non-carbon additives, including metal oxides, LDHs, 2D nanomaterials, MOFs, and metallic nanowires, have demonstrated comparable or even complementary mechanisms. These nanofillers not only tailor the crystalline structure but also serve as charge-trapping centers or conductive pathways, leading to substantial improvements in both electrical output and device stability. Overall, the strategic design of PVDF composites through phase engineering and additive optimization presents a promising route toward high-performance, flexible, and durable TENGs that are suitable for a wide range of self-powering applications. Despite these technological advances, PVDF-based materials have recently become a source of environmental concern. PVDF, a fluorine-containing polymer, is difficult to decompose, which poses an environmental hazard and raises residual issues in terms of sustainable electronic device implementation. Therefore, in the future development of PVDF-based TENGs, a balanced approach that considers not only excellent electrical performance but also environmental responsibility and the possibility of large-area scalability of the process will be necessary.

## 4. Tribo-Positive Materials

Tribo-positive materials, positioned at the positive end of the triboelectric series, are characterized by their tendency to donate electrons when brought into contact with other materials. While often selected for their charge-donating capabilities, recent research has emphasized the importance of their environmental stability. Unlike many tribo-negative materials, tribo-positive materials tend to suffer less from performance degradation under humid or thermally dynamic conditions, making them attractive for commercial applications. Furthermore, certain tribo-positive polymers can be strategically paired with hygroscopic or hydrophilic additives to enhance device output even in humid and moisture-rich environments. For instance, it has been demonstrated that these materials can utilize hydrogen bonding with water molecules to increase the surface positive charge, thereby improving energy harvesting efficiency even under high relative humidity. Additionally, the mechanical durability of tribo-positive materials plays a crucial role in applications that involve frequent friction or repeated mechanical deformation. Tribo-positive materials such as Nylon 66, with high wear resistance, and cellulose, with hydrogen bonding potential and bio-derived sustainability, exemplify how material design can address not only electrical performance but also environmental and mechanical resilience. These insights have prompted a growing number of studies focusing on composite engineering of tribo-positive materials to improve charge retention, environmental tolerance, and overall output performance of TENG devices.

### 4.1. Nylon: Wear-Resistant Polymer

Nylon is one of the most extensively applied tribo-positive polymers in TENGs owing to its high mechanical durability, structural flexibility, and stable surface properties. The excellent resistance to mechanical wear enables stable energy generation during repeated contact–separation or sliding operations. As a polyamide material, nylon contains a large number of polar amide groups, such as –CONH–, which facilitate electron donation and contribute to its position on the positive side of the triboelectric series. The material can be easily fabricated using various processes, such as electrospinning, solution casting, and surface treatment, allowing for broad adaptability in device fabrication. In addition, the thermal and chemical stability of nylon, along with its ability to form hydrogen bonds with functional groups, has made it a promising platform for constructing composite and multilayer TENG architecture. Recent research has focused on enhancing the triboelectric polarity and dielectric properties of nylon using bulk doping, surface functionalization, and incorporation of hybrid fillers. These efforts aim to overcome the limited charge generation capability of unmodified nylon and to realize significant performance improvements.

**Figure 6:**
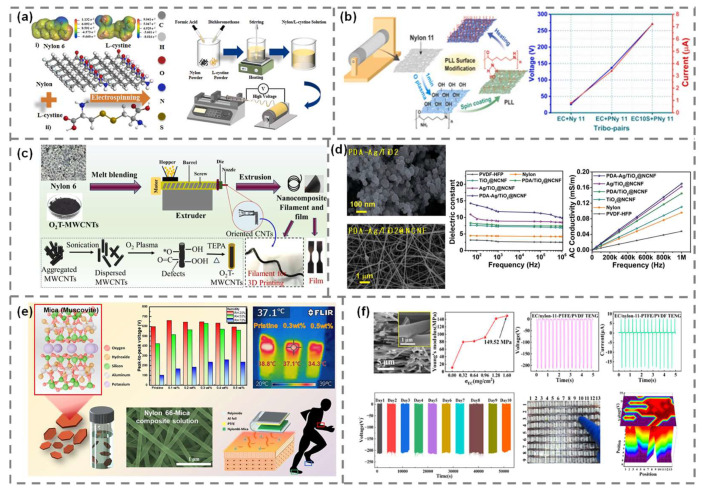
Design strategies for nylon-based composite TENGs. (**a**) Nylon/L-cystine composite nanofiber. Reproduced with permission [64]. Copyright © 2023, Elsevier. (**b**) PLL surface-modified Nylon 11 composite. Reproduced with permission [65]. Copyright © 2023, Elsevier. (**c**) Nylon 6/MWCNT nanocomposite. Reproduced with permission [66]. Copyright © 2024, Elsevier. (**d**) Nylon/PDA-Ag/TiO_2_ nanofiber. Reproduced with permission [67]. Copyright © 2024, WILEY-VCH GmbH, (**e**) Nylon 66/mica composite nanofiber. Reproduced with permission [69]. Copyright © 2024, Elsevier. (**f**) Nylon/fluffy-free EC. Reproduced with permission [70]. Copyright © 2023, American Chemical Society.

Efforts to enhance the tribo-positive characteristics of nylon have focused not only on introducing external fillers but also on modifying the polymer structure itself. One strategy involved blending Nylon 66 with L-cystine and an amino acid featuring strong dipole moments and electron-donating –NH_2_ and –COOH groups. The addition of L-cystine to electrospun nylon fibers resulted in a significantly higher surface charge density due to increased interfacial polarity and hydrogen bonding interactions. A multilayer TENG device fabricated using this composite exhibited a peak output power density of 11.52 W/m^2^, which was more than an order of magnitude higher than that of conventional nylon-based devices (Figure 6a) [64]. This improvement was attributed to both the tribo-positive nature of L-cystine and its molecular compatibility with the nylon matrix. In another approach, surface functionalization of electrospun Nylon 11 was achieved via post-treatment with poly-L-lysine (PLL), a biocompatible polypeptide rich in amine groups (Figure 6b) [65]. The PLL-modified surface introduced positively charged functional groups onto the fiber exterior, enhancing triboelectric charge donation upon contact. As a result, the open-circuit voltage of the TENG increased from 26 to 137 V, and the short-circuit current improved from 0.8 μA to 3.4 μA. By pairing with a SrTiO_3_-doped counter layer, the output voltage and current further increased to 270 V and 7.2 μA, respectively, corresponding to a power density of approximately 2 W/m^2^. These examples demonstrate that molecular-level modulation of the surface of the bulk structure of nylon can significantly enhance its triboelectric polarity and output performance without relying on traditional inorganic or conductive fillers.

Enhancing charge transport and interfacial polarization using functional fillers has been a key strategy for improving the output of nylon-based TENGs. In one study, amin-functionalized MWCNTs were incorporated into a Nylon 6 matrix via solution blending (Figure 6c) [66]. The conductive network formed within the polymer allowed for more efficient charge transport toward the electrodes, while the functional amine groups facilitated strong electrostatic interactions with nylon chains. At an optimized filler loading of 1 wt%, the resulting device produced an open-circuit voltage of 105.7 V and a short-circuit current of 10.5 μA, corresponding to a power density of 465 mW/m^2^. Furthermore, the TENG maintained electrical stability over 20,000 continuous operation cycles without significant degradation, indicating both the electrical and mechanical robustness of the composite structure.

A similar synergistic effect was demonstrated using a hybrid filler consisting of polydopamine-coated Ag-decorated TiO_2_ nanoparticles (Figure 6d) [67]. This composite combined the benefits of a high dielectric constant from TiO_2_ and partial conductivity from the metallic Ag phase. When electrospun with nylon fibers, the hybrid filler significantly enhanced both the dielectric environment and the charge transfer efficiency. The device achieved a maximum open-circuit voltage of approximately 370 V and delivered a power density of 5.4 W/m^2^, significantly surpassing that of pristine nylon or TiO_2_-only counterparts. These results demonstrate that rationally engineered conductive or hybrid fillers can overcome the limited charge transport and interfacial polarization of unmodified nylon, resulting in substantial improvements in TENG performance.

Beyond conductive additives, insulating inorganic nanofillers have been widely used to enhance the dielectric properties and surface charge accumulation of nylon-based triboelectric materials. In one study, TiO_2_ nanoparticles were directly incorporated into electrospun Nylon 66 nanofibers at various weight ratios [68]. The TiO_2_-nylon composite exhibited a significant increase in output with increasing filler content, reaching an optimal concentration of 6.4 wt%. At this loading amount, the device achieved an open-circuit voltage of approximately 120 V and a short-circuit current of 6.9 μA, corresponding to a power density of 1.75 mW/cm^2^. Compared to pristine Nylon 66, the output voltage and power were enhanced by about 25% and 1.9 times, respectively. These improvements were attributed to the increased dielectric constant induced by the TiO_2_ filler, which allowed greater charge separation during contact electrification.

Another approach employed mica, a naturally insulating silicate mineral with high thermal and chemical stability, blended with Nylon 66 to form a humidity-resistant composite nanofiber. The incorporation of mica significantly suppressed the leakage of triboelectric charges, maintaining device performance even under 70% relative humidity (RH) (Figure 6e) [69]. At 20% RH, the optimized composite containing 0.3 wt% mica achieved a peak-to-peak open-circuit voltage of approximately 659 V, showing a notable improvement over pristine Nylon 66. Furthermore, under 50% RH, the same composite delivered a maximum power density of 5.36 mW/cm^2^, more than twice that of pristine Nylon 66. The device also maintained a stable output of over 100,000 operation cycles and successfully powered 80 light-emitting diode arrays without temperature rise or degradation. These enhancements were attributed to the excellent insulation capability and low dielectric loss of mica, which minimized charge dissipation and prevented dielectric breakdown during repeated contact operations.

To improve the mechanical robustness and long-term operational stability of nylon-based TENGs, researchers have explored structural reinforcement through polymer blending. One study introduced ethyl cellulose (EC), a biocompatible and hydrophobic polymer, as a surface coating layer on electrospun Nylon 11 nanofibers (Figure 6f) [70]. The addition of EC produced a fluffy-free morphology that eliminated fiber entanglement and enhanced the mechanical stiffness of the nanofiber mat. Notably, the elastic modulus of the composite increased by approximately 12 times compared to pristine Nylon 11, indicating a significant improvement in structural rigidity. This enhancement not only improved surface contact efficiency but also extended device lifetime under repeated operation. The EC/Nylon 11 TENG exhibited an open-circuit voltage of approximately 212 V, a short-circuit current of 18.5 μA, and a power density of 1.76 W/m^2^ while maintaining consistent performance over 100,000 operating cycles. The device also demonstrated excellent thermal endurance, continuing to operate stably under elevated temperatures without material degradation or signal drift. These results suggest that mechanical reinforcement using thermally stable secondary polymers such as EC can substantially improve both the electrical and mechanical reliability of tribo-positive TENGs for commercial applications.

In summary, nylon serves as a versatile and robust tribo-positive polymer for TENG applications, offering excellent mechanical durability, high processability, and strong tribo-positivity. Recent research has demonstrated that both molecular-level modification and composite engineering can significantly enhance its performance. Improvements in charge generation, charge retention, and long-term mechanical stability have been achieved through a systematic approach to material design and structural engineering. Table 4 summarizes the processing strategies and performance reported in the reviewed studies on nylon-based TENG. These advances not only address the intrinsic limitations of pristine nylon but also extend its applicability to harsh environments involving high humidity, elevated temperatures, and repeated mechanical stress. However, nylon still has the disadvantage of low stability in high-humidity environments compared to other tribo-positive materials such as cellulose. The hygroscopic surface of nylon can absorb moisture, which hinders charge transfer, resulting in a decrease in the output performance of the TENG. This problem becomes more important considering the difficulty in controlling humidity in practical application environments. Therefore, future studies are required to enhance the environmental durability of nylon-based TENGs and enable stable operation under various conditions.

### 4.2. Cellulose: Sustainable and Bio-Derived Polymer

Cellulose is an abundant and sustainable biopolymer, valued for its excellent physical and chemical properties, as well as its inherent biodegradability and biocompatibility [71]. As a triboelectric material, cellulose generally exhibits positive polarity due to its tendency to donate electrons, and its hydroxyl-rich surface chemistry can be readily modified to enhance triboelectric performance. The material can be processed into nanofibers or flexible films, allowing TENG devices to remain lightweight, conformable, and suitable for wearable and deformable applications. Additionally, cellulose shows outstanding compatibility with various nanomaterials and functional fillers, enabling composite structures that enhance charge generation and improve output stability, particularly under humid conditions. These combined features, such as sustainability, modifiable surface chemistry, and mechanical flexibility, make cellulose a highly promising tribo-positive material for eco-friendly and high-performance TENGs.

**Figure 7:**
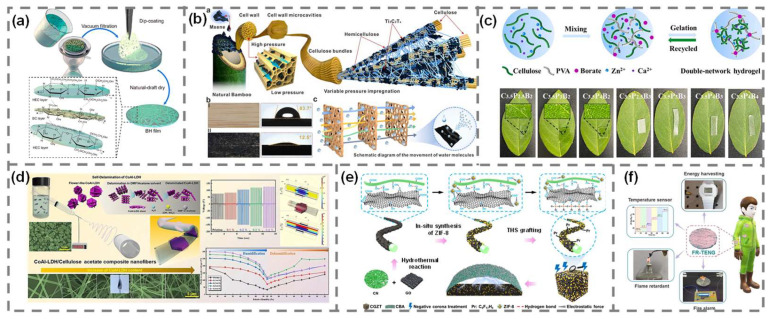
Design strategies for cellulose-based composite TENGs. (**a**) Bacterial cellulose-based TENG. Reproduced with permission [72]. Copyright © 2022, American Chemical Society. (**b**) Cellulose/MXene composite. Reproduced with permission [73]. Copyright © 2023, Elsevier. (**c**) Cellulose/PVA hydrogel. Reproduced with permission [74]. Copyright © 2024, Elsevier. (**d**) Cellulose/CoAl-LDH composite nanofiber. Reproduced with permission [75]. Copyright © 2024, Elsevier. (**e**) Cellulose/graphene oxide/ZIF-8 composite. Reproduced with permission [76]. Copyright © 2024, American Chemical Society. (**f**) CNF-BP-PA composite film. Reproduced with permission [78]. Copyright © 2022, Elsevier.

One effective strategy to enhance the performance of cellulose-based TENGs is to modulate the surface potential and polarity of the tribo-positive layer. In a recent study, bacterial cellulose (BC) was combined with hydroxyethyl cellulose (HEC) to fabricate a nanofiber composite. The composite was prepared by drop-casting an aqueous HEC solution onto a dried BC film, followed by ambient drying, which resulted in a homogeneous coating and infiltration of HEC into the BC nanofibers (Figure 7a) [72]. The introduction of HEC, which contains abundant hydroxyl groups, significantly altered the surface charge characteristics of the composite, thereby enhancing its tribo-positive property. The resulting TENG exhibited notable performance improvements, achieving an open-circuit voltage of 76.61 V, a short-circuit current of 8.68 μA, and a transferred charge of 26.92 nC. These values represented a substantial increase compared to pristine BC. The enhanced output was attributed to improved contact electrification and surface polarization facilitated by molecular interactions between HEC and BC. In addition to improved TENG performance, the device also demonstrated excellent stability and durability. The composite nanofiber maintained a consistent electrical output under repeated 10,000 contact–separation cycles, showing good potential for wearable or motion-sensing applications. The study highlights that simple surface chemistry modification using cellulose derivatives can effectively modulate TENG performance without requiring complex nano-structuring or conductive additives.

Similar to the strategies observed in nylon-based TENGs, the incorporation of conductive nanomaterials into cellulose matrices has proven to be an effective approach for enhancing charge transfer and improving TENG performance. Ti_3_C_2_T_x_ MXene was introduced into a delignified cellulose scaffold via vacuum-assisted impregnation (Figure 7b) [73]. Strong hydrogen bonding between the -OH groups of cellulose and MXene facilitated stable incorporation, while the layered structure of MXene expanded interfacial areas and promoted charge trapping. The resulting TENG demonstrated a peak power density of 25 μW/cm^2^ and an open-circuit voltage of 84 V, representing a 25-fold increase in power output compared to pristine cellulose-based devices. The device also exhibited strong mechanical stability, maintaining consistent output of over 1200 continuous contact and separation cycles without structural degradation. Moreover, the cellulose/MXene composite showed high humidity sensitivity across a range of 40–90% RH, with a sensitivity of 0.8 V/%RH and a fast response time of approximately 150 s. These features make the composite a promising candidate for both TENG and self-powered humidity sensing. As in other tribo-positive polymer systems, the integration of MXene into cellulose matrices enhances surface potential, charge retention, and environmental adaptability, which are beneficial for forming a multifunctional platform for advanced TENG applications.

While the previous cellulose composites primarily focused on enhancing charge generation and surface potential, certain applications require materials with exceptional flexibility, mechanical durability, and environmental stability. Addressing these demands, a double-network (DN) hydrogel composite was developed by combining PVA-borax with cellulose cross-linked by Ca^2+^ and Zn^2+^ ions (Figure 7c) [74]. The optimized condition, denoted C_3.5_P_3_B_2_ (where C, P, and B represent cellulose, PVA, and borax, respectively), exhibited significantly enhanced mechanical properties, with a tensile stress of 159.4 kPa, a tensile strain of 683.5%, and a toughness of 541.5 kJ/m^3^. It also demonstrated strong cyclic fatigue resistance, maintaining stable stress values over 10 consecutive cycles at 100% strain. The hydrogel preserved high transparency and non-drying behavior under ambient storage for 30 days and displayed anti-freezing performance down to −70 ℃ with a maintained ionic conductivity of 1.8 S/m after freezing. Furthermore, recyclability was verified via thermal reprocessing, and the regenerated hydrogel retained comparable mechanical and electrical properties. When integrated into a TENG, the device achieved a maximum open-circuit voltage of 135 V and power density of 1.81 W/m^2^, with stable operation under mechanical deformation and across a broad temperature range (−40 to 50 °C), underscoring its potential in sustainable and self-powered electronics.

To enhance the humidity tolerance of cellulose-based TENGs, three studies employed distinct material strategies and fabrication methods that leveraged unique mechanisms to maintain output performance under high-humidity conditions. In one study, a composite nanofiber was fabricated by electrospinning cellulose acetate (CA) with CoAl-layered double hydroxides (CoAl-LDHs) synthesized via a hydrothermal process (Figure 7d) [75]. The CA nanofibers containing 0.3 wt% CoAl-LDH sheets increased the charge density through their intrinsic positive surface potential and high hydrophilicity, enabling stable hydrogen bonding with ambient moisture. As a result, the composite nanofiber-based TENG exhibited an output voltage of 300 V and current of 4.3 μA under 20% RH, representing 62% and 44% improvements over pristine CA nanofiber, respectively. More importantly, it maintained robust performance up to 95% RH and delivered a power density of 638 μW/cm^2^ under 70% RH, demonstrating superior humidity resistance. Durability testing also showed excellent performance over 10,000 cycles. In the second study, cellulose nanofibers (CN) were mixed with graphene oxide (GO) to form a porous aerogel scaffold, which was used to support the in situ growth of ZIF-8. The resulting structure was further modified by treatment with a fluorinated silane (THS) and exposed to a negative corona discharge (Figure 7e) [76]. The hydrothermally synthesized composites consisting of CN/GO/ZIF-8/THS formed a highly porous and micro-structured film with improved hydrophobicity. This architecture enhanced moisture resistance and helped to stabilize charge retention. Under optimal conditions (GO/CN ratio of 1.5), the device achieved 180 V and 24 μA, with only ~26% loss in PM0.3 filtration efficiency after 2.5 h under 95% RH. While both strategies successfully mitigated humidity-induced charge dissipation, their mechanisms differ fundamentally. The CoAl-LDH/CA composite nanofiber utilized hygroscopic ionic interactions to stabilize the charge density in CA fibers, whereas CGZT relied on surface hydrophobicity. These complementary approaches offer valuable pathways for designing humidity-resistant cellulose-based TENGs. In another study, surface functionalization using amine-rich polymers was an effective way to enhance the TENG performance of tribo-positive materials. Polyethyleneimine (PEI) was introduced onto the surface of cellulose nanofibers to form hydrogen-bonded interfacial layers [77]. This modification significantly improved the surface charge trapping and polarization efficiency by altering the triboelectric potential of the cellulose matrix. As a result, the optimized cellulose/PEI TENG achieved an open-circuit voltage of 157.3 V, a short-circuit current of 3.47 μA, and a peak power density of 0.146 W/m^2^. Beyond electrical performance, the composite film also exhibited a 4.3-fold increase in wet strength, which is crucial for device reliability under high-humidity conditions. These findings underscore that PEI-assisted surface engineering can simultaneously enhance the mechanical properties and TENG performance of cellulose-based TENGs, thereby expanding their applicability to harsh conditions.

Another strategy to enhance the environmental adaptability of cellulose-based TENGs involved the incorporation of flame-retardant additives to improve thermal stability and enable fire-warning capabilities. Cellulose nanofibers (CNF) were combined with tannic acid-stabilized black phosphorus nanosheets (TA-BPNS) and phytic acid (PA) via vacuum filtration to form a CNF-BP-PA composite film. Silver nanowires (AgNWs) were subsequently deposited to serve as the conductive layer, and a single-electrode TENG device was assembled (Figure 7f) [78]. This structure achieved an open-circuit voltage of 116 V and a current density of 3.02 mA/m^2^. The device also exhibited a humidity sensitivity of 1.436 V/%RH and an extremely high thermal sensitivity index of 3779.16 K, allowing it to function as a temperature sensor and fire alarm with a response time of less than 5 s under direct flame or high-temperature conditions. The flame-retardant mechanism was attributed to multiple synergistic effects: the lamellar TA-BPNS structure inhibited heat conduction; P2O5 and other phosphorus-rich compounds formed insulating barriers to block oxygen and thermal diffusion; and PO^•^ and HPO^•^ radicals quenched flame propagation at the molecular level. As a result, the composite film exhibited a limiting oxygen index of 39.1%, and the peak heat release rate and total heat release were reduced by 64.6% and 47.6%, respectively, compared to pure CNF. These results demonstrate that functional filler design in cellulose composite can extend beyond energy harvesting to include flame detection and environmental safety functions, broadening the application potential of TENG technology.

Another promising direction involves the utilization of kapok-derived cellulose nanofibers (KCNF) for TENG applications [79]. Kapok fibers, a natural waste byproduct, were TEMPO-oxidized and mechanically treated to fabricate flexible and transparent KCNF films with high dielectric properties and enhanced surface potential. The resulting KCNF exhibited a high transmittance (>90%) and excellent tensile strain (~25%), making it well suited for skin-mounted wearable devices. When employed in a TENG structure using Ag nanowires as the conductive layer, the KCNF-based device demonstrated significant output enhancement, achieving an open-circuit voltage of 75 V, a short-circuit current of 3.8 μA, and a peak power density of 0.8 W/m^2^. The improved performance was attributed to the denser, smoother surface morphology and increased dielectric constant obtained via mechanical treatment, which enhanced both contact electrification and charge retention. Beyond energy harvesting, the KCNF-TENG exhibited robust biocompatibility and degradability, retaining its structural integrity under acidic, basic, and humid conditions, while degrading by over 98% within 10 days in natural soil. These results underscore the effectiveness of structural and dielectric tuning in kapok cellulose-based composites for enhancing TENG performance while maintaining environmental sustainability.

To further improve energy harvesting and multifunctionality in cellulose-based TENGs, a flexible device was developed using electrospun cellulose acetate (ES-CA) as the tribo-positive layer and a boron nitride nanosheet-polyvinylpyrrolidone (BN-PVP) composite as the tribo-negative layer [80]. With a 9 cm^2^ contact area and a 19 mm separation gap, the resulting TENG achieved an open-circuit voltage of approximately 1200 V, a short-circuit current of 1.2 mA, and a peak power density of 1.4 W/m^2^. Notably, this performance was over 100-times greater than that of a comparable PVP/ES-CA TENG without BN fillers. Furthermore, the device exhibited excellent flexibility and long-term durability, demonstrating its viability for wearable applications. Beyond energy harvesting, the same materials were adapted to create a self-powered tactile sensor capable of detecting forces as low as 0.05 N, with a sensitivity of 3.98 V/N, highlighting the multifunctional potential of BN-integrated cellulose composites in flexible and autonomous electronics.

In summary, cellulose has attracted attention as one of the most sustainable and eco-friendly materials for TENG applications due to its excellent biodegradability. As a natural polymer, its abundant resources and renewability make it a highly valuable core material for eco-friendly energy harvesting technology. However, compared to other tribo-positive polymers, such as nylon, cellulose-based TENGs generally exhibit lower electrical output. This limitation is due to two factors: the inherent low tribo-positivity of cellulose and the limited processability of its native form. In particular, cellulose is difficult to process into nanofibers through the electrospinning process; therefore, derivatives such as cellulose acetate are typically used to achieve the desired fiber shape. Although these derivatives enhance processability, they can also cause the degradation of TENG performance or dielectric properties. Nevertheless, the unique combination of biodegradability, flexibility, and multifunctionality makes cellulose a promising material platform for the development of next-generation TENGs for sustainable and wearable applications.

In conclusion, this review provided a comprehensive overview of polymer composite-based TENGs, emphasizing material strategies tailored to triboelectric polarity. Recent advances in tribo-negative polymers, such as PDMS and PVDF, have demonstrated that incorporating functional nanofillers can effectively enhance dielectric properties, surface charge density, and environmental durability. For tribo-positive polymers, such as nylon and cellulose, composite engineering has proven to be a versatile approach for improving triboelectric polarity, mechanical robustness, and operational stability under diverse conditions. Notably, the integration of bio-derived and multifunctional materials, particularly cellulose, has enabled the development of TENGs capable of both energy harvesting and environmental sensing. These findings collectively underscore the importance of material innovation in overcoming the inherent limitations of traditional polymers and in expanding the functional scope of TENGs. Future research should aim to advance scalable fabrication methods, enhance long-term durability, and integrate multifunctionality, thereby accelerating the practical deployment of TENG technology in wearable, portable, and sustainable energy systems.

## 5. Perspectives or Challenges

Over the past decade, research on polymer composite-based TENGs has made remarkable strides, with significant improvements in energy output, mechanical flexibility, and functional versatility. Table 5 summarizes process and performance reported in the reviewed studies on cellulose-based composite TENG. These advantages make polymer composite-based TENGs particularly suitable for next-generation applications such as wearable electronics, implantable biomedical systems, and Internet of Things (IoT) devices, where lightweight, flexible, and miniaturized energy harvesting solutions are essential. However, each of these application domains presents unique material challenges that must be addressed for practical implementation. For wearable and textile-integrated systems, maintaining mechanical robustness under repeated deformation and washing cycles is crucial. Implantable biomedical devices demand biocompatible and sterilizable materials with stable output in moist or biofluid-rich environments. IoT sensor nodes often operate in outdoor or industrial settings, where environmental resistance to humidity, temperature fluctuations, and long-term mechanical fatigue becomes essential. Looking ahead, several key areas deserve further attention to advance the practical applications of these devices. First, boosting energy density and overall output remains essential to support higher power demands. Strategies such as integrating high-dielectric fillers, optimizing polymer–filler interfaces, and developing hierarchical structures that facilitate charge transfer can help to achieve this goal. Second, enhancing long-term stability is crucial for real-world use. Polymer composites can degrade in humid or high-temperature environments or under repeated mechanical stress. Future work should focus on incorporating hydrophobic coatings, self-healing polymers, or other robust materials to ensure consistent performance. Third, multifunctionality is a promising direction for polymer composite-based TENGs. By tailoring the composite structure or adding functional nanomaterials, TENGs can be engineered to perform additional roles, such as sensing motion, temperature, or environmental changes, alongside energy harvesting. This could open doors for smart textiles, health monitoring systems, and other integrated applications. Fourth, scalable and cost-effective fabrication methods are crucial for transitioning polymer composite-based TENGs from laboratory research to commercial applications. However, as device dimensions increase, new challenges arise that do not significantly affect small-scale devices. In particular, it becomes increasingly difficult to maintain effective contact between frictional surfaces, which is essential for efficient charge generation. For roll-to-roll processing, maintaining a uniform layer thickness and consistent micro- and nano-patterning over large areas is critical, as even slight deviations can result in reduced surface contact effectiveness and, consequently, diminished output performance. Similarly, in 3D printing, factors such as layer resolution, surface roughness, and shape fidelity can introduce inconsistencies that impair intimate contact between triboelectric layers. Ensuring that large-area devices achieve comparable surface interaction efficiency as their small-scale counterparts is a key scalability hurdle. Finally, effective integration with other systems, such as energy storage devices and wireless communication modules, will be essential to realize practical self-powered systems. However, the pulsed and high-impedance nature of TENG output poses significant challenges when interfacing with storage systems, such as supercapacitors or batteries. These mismatches often require auxiliary power management units, including rectifiers and impedance-matching circuits, to enable efficient energy transfer and storage. To address this, recent efforts have focused on developing DC-TENG and reducing internal resistance to ensure more continuous and compatible power output. Future research should emphasize co-designing integrated modules with optimized electrical interfaces to enhance energy conversion, storage, and utilization in commercial applications.

In summary, building on the progress achieved so far, future research on polymer composite-based TENGs should focus on enhancing energy output, improving stability, incorporating multifunctionality, developing scalable manufacturing methods, and ensuring seamless system integration. Addressing these challenges will help to unlock the full potential of polymer composite-based TENGs in powering the next generation of self-sustaining electronic systems.

## Figures and Tables

**Table 1:** TENG performance of PDMS-based composites.

Composite Materials	Process	Performance (Surface Area)	References
**PDMS/SrTiO_3_**	Spin-coating	130 V_OC_, 1.4 μA, 90 μW	[38]
**PDMS/BNNO-MWCNTs**	Casting	300 V_OC_, 8.5 μA, 7.03 W/m^2^ ($2\times 2\;{cm}^{2}$)	[39]
**PDMS/Silica**	Blade Coating	254 V_OC_, 20.4 μA, 4.37 W/m^2^	[40]
**PDMS/AgNWs**	Casting and Rolling	33.4 V_OC_, 4.5 mA, 0.162 W/m^2^ ($4\times 5\;{cm}^{2}$)	[41]
**PDMS (Agent ratio)**	Spin-coating	54.3 V_OC_ ($1\times 1\;{cm}^{2}$)	[42]
**PDMS/MXene**	Spin-coating	453 V_OC_, 131 μA	[43]

**Table 2:** Performance enhancement strategy of PVDF-carbon composites-based TENGs. ↑ indicates an increase in the corresponding performance enhancement factor compared to pristine PVDF.

Composite Materials	Process	Performance Enhancement Factor	Performance (Surface Area)	References
**PVDF/Graphene QDs**	Electrospinning	*β*-phase fraction ↑	27 mW/m^2^ ($6\times 6\;{cm}^{2}$)	[51]
**PVDF/Graphene Nanosheet**	Electrospinning	*β*-phase fraction ↑	1511 V_OC_, 189 mA/m^2^, 130.2 W/m^2^ ($2\times 2\;{cm}^{2}$)	[52]
**PVDF/Graphene**	Electrospinning	*β*-phase fraction ↑	200 V_OC_, 16.3 μA, 11.32 W/m^2^ ($3\times 4\;{cm}^{2}$)	[53]
**PVDF/Graphene Oxide**	Blade Coating	*β*-phase fraction ↑ Permittivity ↑	318.5 V_OC_, 2.1 μA/cm^2^, 2.6 W/m^2^ ($4\times 4\;{cm}^{2}$)	[54]
**PVDF/Graphene Nanoplatelet**	Casting and Rolling	*β*-phase fraction ↑	28.4 V_OC_, 30 μA, 53.57 mW/m^2^ ($8\;{cm}^{2}$)	[55]
**PVDF-HFP/Graphene**	Electrospinning	Permittivity ↑	1024 V_OC_, 1.11 μA/cm^2^, 1.95 W/m^2^	[56]

**Table 3:** TENG performance of PVDF/non-carbon-additive-based composites.

Composite Materials	Process	Performance (Surface Area)	References
**PVDF/BaTiO_3_**	Electrospinning	307 V_OC_, 1.8 μA/cm^2^, 11.2 W/m^2^ ($2\times 2\;{cm}^{2}$)	[57]
**PVDF/Fe_3_O_4_**	Electrospinning	138 V_OC_, 5.68 μA ($5\times 5\;{cm}^{2}$)	[58]
**PVDF/ZnAl LDH**	Spin-coating	230.6 V_OC_, 5.6 μA, 4.3 W/m^2^ ($3\times 3\;{cm}^{2}$)	[59]
**PVDF/MXene**	Electrospinning	0.228 W/m^2^ ($4\times 4\;{cm}^{2}$)	[60]
**PVDF/MoS_2_**	Electrospinning	208 V_OC_, 31 μA, 1.42 W/m^2^ ($3\times 3\;{cm}^{2}$)	[61]
**PVDF/MOFs(MIL-101)**	Electrospinning	536 V_OC_, 21.7 μA, 5.68 W/m^2^ ($2\times 2\;{cm}^{2}$)	[62]
**PVDF/Ag NWs**	Electrospinning	240 V_OC_, 12 μA ($2\times 2\;{cm}^{2}$)	[63]

**Table 4:** TENG performance of nylon-based composites.

Composite Materials	Process	Performance (Surface Area)	References
**Nylon/L-cystine**	Electrospinning	325 V_OC_, 50 μA, 10.26 W/m^2^ ($3\times 3\;{cm}^{2}$)	[64]
**Nylon 11/Poly-L-lysine(PLL)**	Electrospinning	270 V_OC_, 7.2 μA, 2 W/m^2^ ($2\times 2\;{cm}^{2}$)	[65]
**Nylon 6/MWCNTs**	Hot press compression molding	105.7 V_OC_, 10.55 μA, 0.465 W/m^2^ ($2\times 2\;{cm}^{2}$)	[66]
**Nylon 66/PDA-Ag/TiO_2_ NP**	Electrospinning	370 V_OC_, 15 μA, 5.4 W/m^2^ ($2\times 2\;{cm}^{2}$)	[67]
**Nylon 66/TiO_2_ NPs**	Electrospinning	506 V_OC_, 21.8 μA, 17.5 W/m^2^ ($1\times 1\;{cm}^{2}$)	[68]
**Nylon 66/Mica**	Electrospinning	659 V_OC_, 53.6 W/m^2^ ($2\times 2\;{cm}^{2}$)	[69]
**Nylon 11/Fluffy-Free EC**	Electrospinning	212 V_OC_, 18.5 μA, 1.76 W/m^2^ ($2.5\times 2.5\;{cm}^{2}$)	[70]

**Table 5:** TENG performance of cellulose-based composites.

Composite Materials	Process	Performance (Surface Area)	References
**Bacterial Cellulose/** **Hydroxyethyl Cellulose**	Dip-coating/Drying	76.6 V_OC_, 8.68 μA, 0.72 W/m^2^ ($2\times 2\;{cm}^{2}$)	[72]
**Cellulose/MXene**	Impregnation	84 V_OC_, 0.25 W/m^2^ ($4\times 1\;{cm}^{2}$)	[73]
**Cellulose/PVA Hydrogel**	Casting	211.3 V_OC_, 1.81 W/m^2^ ($2\times 2\;{cm}^{2}$)	[74]
**Cellulose Acetate/CoAl-LDH**	Electrospinning	160 V_OC_, 6.38 W/m^2^ ($1\times 1\;{cm}^{2}$)	[75]
**Cellulose/Graphene Oxide/** **ZIF-8**	Hydrothermal synthesis Freeze-drying THS surface modification	180 V_OC_, 24 μA, 0.413 W/m^2^	[76]
**Cellulose/PEI**	Pulping	157.3 V_OC_, 3.47 μA, 0.146 W/m^2^ ($5\times 5\;{cm}^{2}$)	[77]
**Cellulose/** **Black Phosphorus/Phytic Acid**	Vacuum Filtration	116 V_OC_, 3.8 μA, 0.114 W/m^2^	[78]
**Kapok Cellulose**	TEMPO-Oxidation	75 V_OC_, 3.8 μA, 0.8 W/m^2^ ($2\times 2\;{cm}^{2}$)	[79]
**Cellulose/2D Boron Nitride**	Electrospinning	1200 V_OC_, 1.2 mA, 1.4 W/m^2^ ($3\times 3\;{cm}^{2}$)	[80]

## Data Availability

No new data were created or analyzed in this study.
